# The Role of Non-Coding Regions in Breast Cancer: From Gene Regulation to Therapeutic Implications

**DOI:** 10.3390/ph18091370

**Published:** 2025-09-12

**Authors:** Hussein Sabit, Sara Sobhy, Shaimaa Abdel-Ghany, Al-Hassan Soliman Wadan, Olubukola Ayodele, Yasser Albrahim, Hirendra N. Banerjee, Ahmed Elhashash, Borros Arneth

**Affiliations:** 1 Department of Medical Biotechnology, College of Biotechnology, Misr University for Science and Technology, P.O. Box 77, Giza 3237101, Egypt; 2 Molecular Pathology Laboratory, Children Cancer Hospital Egypt-57357, Cairo 4260102, Egypt; 3 Department of Environmental Biotechnology, College of Biotechnology, Misr University for Science and Technology, P.O. Box 77, Giza 3237101, Egypt; 4 Oral Biology Department, Faculty of Dentistry, Galala University, Galala Plateau, Attaka, Suez 15888, Egypt; 5 University Hospitals of Leicester NHS Trust, Leicester LE1 5WW, UK; 6 Ministry of Health, Alahsa 39182, Saudi Arabia; 7 Department of Natural, Pharmacy and Health Sciences, Elizabeth City State University Campus of the University of North Carolina, Elizabeth City, NC 27909, USA; 8 Institute of Laboratory Medicine and Pathobiochemistry, Molecular Diagnostics, Hospital of the Universities of Giessen and Marburg (UKGM), Philipps University Marburg, Baldinger Str., 35043 Marburg, Germany; 9 Institute of Laboratory Medicine and Pathobiochemistry, Molecular Diagnostics, Hospital of the Universities of Giessen and Marburg (UKGM), Justus Liebig University Giessen, Feulgen Str., 35392 Giessen, Germany

**Keywords:** BC, non-coding DNA, gene regulation, tumor development, therapeutic strategies

## Abstract

Breast cancer (BC) remains one of the most prevalent cancers worldwide and a significant cause of cancer-related mortality among women. Despite significant advancements in understanding the genetic foundations of BC, numerous research initiatives have historically focused on protein-coding genes, which constitute merely about 2% of the human genome. This focus has produced significant insights into oncogenes such as HER2, TP53, and BRCA1, along with tumor suppressor genes. Nonetheless, it has led to the non-coding portions of the genome garnering relatively less focus. Recent studies illuminate the crucial significance of non-coding DNA in cancer biology, highlighting its regulatory roles and influence on tumor formation, metastasis, and treatment resistance. This review examines the importance of non-coding DNA in BC. It provides an in-depth analysis of essential non-coding regions, their functions in gene regulation and chromatin structure, and their implications for various BC subtypes. Examining these facets, we seek to reveal the potential of non-coding DNA as a viable source of novel diagnostic markers and treatment approaches.

## 1. Introduction

Breast cancer (BC) remains a significant global health challenge, characterized by its prevalence and molecular complexity [1,2,3]. While significant progress has been made in early detection and targeted therapies, therapeutic resistance, and disease progression continue to pose formidable clinical obstacles [4]. Traditionally, cancer research has predominantly focused on coding mutations, overlooking the vast potential of the non-coding genome [5,6]. Recent studies are beginning to highlight the importance of non-coding regions, as they play critical roles in gene regulation and may harbor mutations that contribute to cancer development and resistance [5].

Once dismissed as junk DNA, the non-coding genome is now recognized as a rich repository of regulatory components that govern gene expression, chromatin structure, and cellular identity.

**Figure 1:**
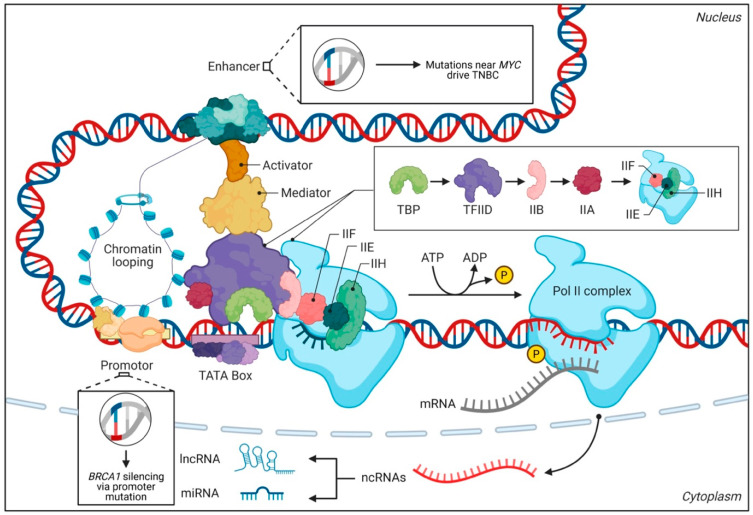
Transcriptional regulation and non-coding mutations in BC. This figure illustrates the complex molecular mechanisms of gene regulation and transcription in eukaryotic cells, highlighting how non-coding DNA elements influence cancer development. Non-coding DNA encompasses diverse elements such as enhancers, promoters, silencers, and ncRNAs. These elements exert precise control over gene expression by modulating transcription, RNA splicing, and chromatin organization. The diagram shows DNA with various regulatory elements and protein complexes involved in transcription. Enhancers, which are distal regulatory sequences, interact with promoters through chromatin looping to enhance transcription, as visualized in the top portion of the figure. The central area shows the assembly of the transcriptional apparatus, including the activator protein (brown), mediator complex (light tan), and promoter region with a TATA box motif. The right side displays the sequential assembly of basal transcription factors (TBP, TFIID, IIB, IIA, IIE, IIF, and IIH) forming the pre-initiation complex, leading to RNA Polymerase II (Pol II) activity. In BC, mutations within these non-coding regions can disrupt intricate regulatory networks, as illustrated by the two highlighted examples: mutations near the *MYC* oncogene that drive its overexpression in TNBC, contributing to tumor aggressiveness and chemoresistance, and mutations in the promoter region of *BRCA1* resulting in its silencing, which impairs DNA repair mechanisms and fosters genomic instability. The bottom portion shows transcription products entering the cytoplasm, including mRNA and ncRNAs that branch into long non-coding RNAs (lncRNAs) and microRNAs (miRNAs), which regulate gene expression at both transcriptional and post-transcriptional levels, influencing critical cellular processes like proliferation, differentiation, and apoptosis.

Approximately 98% of the human genome comprises non-coding DNA, encompassing diverse elements such as enhancers, promoters, silencers, and non-coding RNAs (ncRNAs) [7]. These elements exert precise control over gene expression by modulating transcription, RNA splicing, and chromatin organization. Enhancers, distal regulatory sequences, interact with promoters through chromatin looping to enhance transcription [8]. Similarly, ncRNAs, including long non-coding RNAs (lncRNAs) and microRNAs (miRNAs), regulate gene expression at both transcriptional and post-transcriptional levels, influencing critical cellular processes like proliferation, differentiation, and apoptosis [9]. In BC, mutations within these non-coding regions can disrupt intricate regulatory networks, leading to the aberrant expression of oncogenes and tumor suppressor genes. For example, mutations in enhancers proximal to the *MYC* oncogene drive its overexpression in triple-negative breast cancer (TNBC), contributing to tumor aggressiveness and chemoresistance [10]. Likewise, mutations in the promoter region of tumor suppressor genes, such as *BRCA1*, can result in their silencing, impairing DNA repair mechanisms and fostering genomic instability [11] (Figure 1). These findings underscore the profound impact of non-coding mutations on the transcriptional landscape of BC, emphasizing the necessity for a comprehensive understanding of their functional consequences.

**Figure 2:**
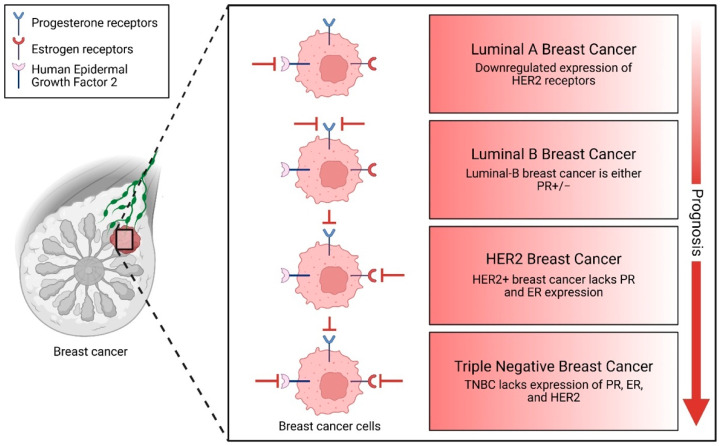
BCs are commonly classified into several subtypes based on the expression of hormone receptors and HER2. Luminal A BC is characterized by the absence of HER2 expression. Luminal B BC may be either positive or negative for progesterone receptors (PR). HER2-positive BC typically lacks both progesterone (PR) and estrogen receptor (ER) expression. TNBC is defined by the absence of all three markers: PR, ER, and HER2.

BC is a highly heterogeneous disease characterized by distinct molecular subtypes, including luminal A, luminal B, HER2-enriched, and basal-like/TNBC [12,13] (Figure 2). Each subtype is shaped by unique genetic and epigenetic processes, with non-coding mutations playing a pivotal role in delineating their specific characteristics and influencing therapeutic responses [14,15,16]. In luminal breast tumors, characterized by estrogen receptor (ER) and/or progesterone receptor (PR) expression, mutations in enhancers and promoters of hormone-responsive genes, such as *FOXA1* and *ESR1*, can disrupt hormone signaling, leading to endocrine therapy resistance [17,18]. HER2-enriched subtypes, driven by *HER2* amplification, harbor non-coding mutations in enhancers and promoters that overactivate signaling pathways, contributing to poor prognosis [19,20]. Basal-like/TNBC, lacking ER, PR, and HER2 expression, exhibits non-coding mutations in enhancers, such as those near MYC, and lncRNAs, such as MALAT1 and NEAT1, which promote stemness, immune evasion, and chemoresistance [21,22,23]. The ability to target these non-coding elements, such as by using BET inhibitors to disrupt enhancers or targeting lncRNAs like *HOTAIR*, offers promising therapeutic strategies to overcome resistance and improve patient outcomes [24,25]. These examples highlight the critical role of non-coding mutations in shaping the biology of BC subtypes and their potential as biomarkers and therapeutic targets.

This review comprehensively explores the functional consequences of non-coding mutations in breast cancer (BC). We elucidate how these mutations interfere with regulatory networks controlling gene expression, chromatin structure, and cellular identity, thereby contributing to tumor heterogeneity and aggressiveness. By understanding the functional ramifications of these mutations, we can identify novel biomarkers and therapeutic targets. Integrating non-coding mutation analysis into clinical practice represents a significant advancement with the potential to revolutionize BC diagnosis, treatment, and management, ultimately improving patient outcomes.

## 2. Key Non-Coding Elements and Their Regulatory Functions

### 2.1. Enhancers and Super-Enhancers

Enhancers are distal regulatory elements that significantly boost the transcription of target genes by interacting with their promoters, often through chromatin looping [26,27]. This looping brings enhancers closer to promoters, facilitating the recruitment of transcription factors and the RNA polymerase machinery. In cancer, particularly BC, the dysregulation of enhancers can lead to aberrant gene expression, a hallmark of the disease. Super-enhancers, clusters of enhancers that drive the expression of oncogenes, have been implicated in BC progression [28]. Studies have shown that super-enhancers are crucial for the overexpression of oncogenes like ERBB2, which is associated with aggressive BC subtypes [29]. Recent research highlights the potential of targeting super-enhancers as a therapeutic strategy in the cancer [30].

In the evolving landscape of cancer research, the intricate roles of non-coding RNAs (ncRNAs) have emerged as a central area of inquiry, moving beyond their initial perception as transcriptional noise. A series of recent studies collectively highlights their profound and multifaceted impact on tumorigenesis, particularly in BC. These ncRNAs are not mere bystanders; rather, they are active participants in fundamental cellular processes, including DNA repair, gene expression regulation, and shaping the tumor microenvironment. A nuanced understanding of their mechanisms is essential for identifying new biomarkers and therapeutic targets.

A compelling and recurring theme within this research is the dual nature of ncRNAs, which can function as either tumor suppressors or oncogenic drivers, depending on the specific context. For instance, some miRNAs act as potent tumor suppressors. Recent research [31] has elegantly demonstrated this by showing that microRNA-1 (miR-1) suppresses breast cancer progression by downregulating the oncogene K-RAS and the long lncRNA MALAT1. This action effectively curtails cancer cell proliferation and promotes apoptosis. Similarly, miR-146a has been identified as another tumor-suppressive miRNA that inhibits breast cancer cell proliferation by directly targeting the well-known cell cycle regulator CDKN2A [32].

Conversely, other ncRNAs actively promote cancer progression. A critical insight into this has been provided by focusing on lncRNA LINP1 in the TNBC [33]. Their work revealed that LINP1 acts as a pro-tumorigenic factor, serving as a scaffold protein that enhances DNA double-strand break repair via the non-homologous end joining (NHEJ) pathway. This protective mechanism makes cancer cells less susceptible to radiation therapy, a crucial finding for optimizing treatment strategies. This oncogenic potential is further illuminated with other study on lncRNA CDKN2B-AS1 [34]. They discovered that this lncRNA sponges miR-122-5p, thereby upregulating the expression of the protein kinase STK39. This intricate regulatory axis involving different ncRNA species highlights the complexity of these networks.

These studies also underscore the intricate crosstalk between ncRNAs and established oncogenes and tumor suppressors. A recent study [35] offered a comprehensive review of the complex interplay between the tumor suppressor protein p53 and ncRNAs in regulating epithelial–mesenchymal transition (EMT), a critical process for metastasis. Interestingly, the authors found that the mutational status of p53 is a key determinant of these interactions, suggesting a deeply integrated regulatory system. While not focused on breast cancer, the work by Zhai et al. [36] on pancreatic cancer offers a compelling model for how ncRNAs can amplify oncogenic signaling. Their study revealed a positive feedback loop where lncRNA LINC01420 boosts the expression of the oncogene MYC, which in turn upregulates KRAS transcription.

Ultimately, these findings are transforming our approach to cancer by pointing to the significant potential of ncRNAs as novel biomarkers and therapeutic targets. A recent article is particularly promising in this regard [37]. Their comprehensive analysis of CDKN2A-correlated genes led to the development of a prognostic model for TNBC, offering a potential pathway toward more personalized medicine. The authors even used this model to propose new drug targets, underscoring the clinical relevance of ncRNA research. The potential for targeting ncRNAs to overcome therapeutic resistance is further highlighted by the identification of LINP1 as a target to enhance radiosensitivity in TNBC and the suggestion that inhibiting the oncogenic lncRNA KIMAT1 could be a viable strategy to block KRAS-driven tumorigenesis [38]. The detailed mechanistic insights from these studies are paving the way for innovative diagnostic tools and targeted therapies, offering a renewed sense of hope for improving cancer patient outcomes.

Table 1 summarizes the diverse roles of lncRNAs, miRNAs, circRNAs, snoRNAs, and piRNAs in BC pathobiology.

### 2.2. Introns

Introns, non-coding sequences within genes, are spliced out during mRNA processing [39,40]. Contrary to their historical neglect, introns harbor regulatory elements such as cryptic promoters and enhancers. Mutations within introns can disrupt normal splicing, producing aberrant protein isoforms associated with cancer. Intronic mutations can create novel splice sites or alter splicing patterns, resulting in truncated or misfunctional proteins [41]. Recent studies have identified intronic variants contributing to cancer pathogenesis by affecting splicing and gene expression regulation [42].

### 2.3. Long Non-Coding RNAs (lncRNAs)

Long non-coding RNAs (lncRNAs) are transcripts longer than 200 nucleotides that do not encode proteins but play critical roles in gene regulation. They function as molecular scaffolds, decoys, or guides, influencing chromatin remodeling, transcription, and post-transcriptional regulation [9]. In BC, dysregulated lncRNAs such as HOTAIR and MALAT1 have been implicated in metastasis, therapy resistance, and poor prognosis. HOTAIR, for instance, is involved in promoting the epithelial-to-mesenchymal transition (EMT), a process crucial for cancer invasion and metastasis [43]. Recent research underscores the potential of lncRNAs as therapeutic targets in BC [44].

### 2.4. MicroRNAs (miRNAs)

MicroRNAs (miRNAs) are small, non-coding RNAs (~22 nucleotides) that post-transcriptionally regulate gene expression by binding the 3′ untranslated regions (UTRs) of target mRNAs, leading to mRNA degradation or translational repression [45]. In BC, aberrant expression of miRNAs, such as the oncogenic miR-21 and the tumor-suppressive miR-200 family, has been linked to cancer pathogenesis, including EMT and invasion. For example, miR-21 is overexpressed in BC and promotes cancer cell proliferation and survival by targeting tumor suppressor genes [46]. Recent studies highlight the potential of miRNAs as biomarkers and therapeutic targets in BC [47].

**Table 1:** Diverse Roles of lncRNAs, miRNAs, circRNAs, snoRNAs, and piRNAs in BC Pathobiology.

Type of ncRNA	Example	Functional Role in BC	Implications	References
**Long Non-Coding RNAs (lncRNAs)**	HOTAIR	Promotes chromatin remodeling via interaction with PRC2; silences tumor suppressor genes.	Drives metastasis and therapy resistance in luminal and TNBC subtypes.	[43]
	MALAT1	Regulates alternative splicing and gene expression; enhances chemoresistance.	Associated with aggressive tumor behavior and poor prognosis in TNBC.	[48]
	NEAT1	Facilitates nuclear paraspeckle formation; regulates DNA repair and cell cycle progression.	Promotes tumor growth and chemoresistance in TNBC.	[49]
	PVT1	Interacts with MYC to upregulate PD-L1; promotes immune evasion.	Enhances immune evasion and resistance to immunotherapy in basal-like/TNBC.	[50]
	XIST	Regulates X-chromosome inactivation; modulates chromatin structure.	Implicated in tumor progression and therapy resistance in luminal and TNBC subtypes.	[51]
**MicroRNAs (miRNAs)**	miR-21	Targets tumor suppressors (PTEN, PDCD4); promotes oncogenic signaling.	Drives tumor growth and chemoresistance in TNBC and HER2-enriched subtypes.	[52]
	miR-200 family	Inhibits EMT by targeting ZEB1/ZEB2; suppresses metastasis.	Downregulated in TNBC; restoration inhibits EMT and reduces tumor aggressiveness.	[53]
	miR-125a/b	Targets HER2; inhibits oncogenic signaling.	Downregulated in HER2-positive BCs; restoration reduces HER2 levels and tumor growth.	[54]
	miR-34a	Regulates cell cycle and apoptosis; targets MYC and BCL2.	Downregulated in TNBC, restoration induces apoptosis and unsensitizeses tumors to therapy.	[55]
	miR-205	Targets HER2 and other oncogenic pathways; inhibits tumor growth.	Downregulated in TNBC; restoration reduces tumor aggressiveness.	[56]
**Circular RNAs (circRNAs)**	circTADA2A	Acts as a miRNA sponge; regulates gene expression.	Promotes tumor growth and metastasis in TNBC.	[57]
	circHER2	Derived from the HER2 gene, it regulates HER2 signaling.	Associated with HER2-positive BCs; potential biomarker for therapy response.	[58]
	circSMARCA5	Regulates alternative splicing and gene expression.	Downregulated in TNBC, restoration inhibits tumor growth and metastasis.	[59]
**Small Nucleolar RNAs (snoRNAs)**	SNORD50A/B	Regulates RNA processing and modification.	Deletions associated with poor prognosis in luminal BCs.	[60]
	SNHG1	Acts as a scaffold for chromatin-modifying enzymes.	Promotes tumor growth and therapy resistance in TNBC.	[61]
**Piwi-Interacting RNAs (piRNAs)**	piR-823	Regulates DNA methylation and gene silencing.	Overexpressed in TNBC; promotes tumor growth and chemoresistance.	[62]

### 2.5. A Multi-Omic Approach to Prognostic and Therapeutic Stratification in Breast Cancer

The molecular landscape of breast cancer is complex, and recent research has increasingly leveraged multi-omic and computational approaches to move beyond simple pathological staging toward personalized prognostic and therapeutic strategies. A significant body of work has successfully identified predictive signatures based on non-coding RNAs and other molecular markers. For instance, multiple studies have developed prognostic models using long non-coding RNA (lncRNA) signatures, which can accurately predict overall patient survival [63,64]. These signatures, remarkably, are independent of traditional clinical factors like age and tumor subtype, allowing for the stratification of patients into high- and low-risk groups with profoundly different survival outcomes. The functional importance of these lncRNAs is further underscored by their connection to critical cancer pathways, such as the cellular response to hypoxia, and their correlation with immune cell infiltration, indicating a direct role in shaping the tumor microenvironment [63]. Parallel investigations into the proteomics of breast cancer have revealed that members of the matrix metalloproteinase (MMP) family, notably MMP1 and MMP9, are differentially expressed. A comprehensive bioinformatics analysis has pinpointed these as potential prognostic markers and therapeutic targets, given their strong correlation with patient survival and the presence of specific immune cell populations [65]. These collective findings highlight the intricate and multifaceted molecular interactions that drive breast cancer progression, emphasizing the critical need for a holistic, integrated view of the disease.

Building on these foundational insights, the integration of AI and machine learning has enabled the creation of truly sophisticated, integrated prognostic tools. A prime example is the Metastatic and Immunogenomic Risk Score (MIRS), a neural network-based system that synthesizes transcriptomics data to simultaneously evaluate both metastatic potential and the tumor immune microenvironment [66,67]. This groundbreaking approach offers a more nuanced stratification of breast cancer patients than was previously achievable, providing not only accurate prognostic predictions but also insights into a patient’s probable response to specific therapies. The MIRS system, for example, has demonstrated that patients in the high-risk group tend to respond more favorably to chemotherapy, whereas those in the low-risk group show a better response to immunotherapy [67]. This provides a robust, data-driven framework for guiding highly individualized treatment plans. These findings are further complemented by network-based studies that have successfully identified “linchpin survival genes” in aggressive subtypes like TNBC, thereby significantly enhancing our ability to prognosticate and potentially target these challenging diseases [68]. Ultimately, this research signifies a profound paradigm shift in oncology, moving decisively away from reliance on single-marker analysis toward integrated, multi-omic, and computational strategies that offer unprecedented precision in understanding and treating breast cancer.

## 3. The Interplay Between Non-Coding DNA and Chromatin Organization

### 3.1. Chromatin Accessibility and 3D Genome Architecture

Non-coding DNA elements, such as enhancers and promoters, play a critical role in regulating gene expression by influencing chromatin accessibility and the three-dimensional organization of the genome [69]. These elements are often located within open chromatin regions characterized by specific histone modifications that mark their activity. Active enhancers are typically marked by H3K27ac (histone H3 lysine 27 acetylation), while active promoters are associated with H3K4me3 (histone H3 lysine 4 trimethylation). These modifications create a permissive chromatin environment, allowing transcription factors and other regulatory proteins to access DNA and initiate gene expression [70].

**Figure 3:**
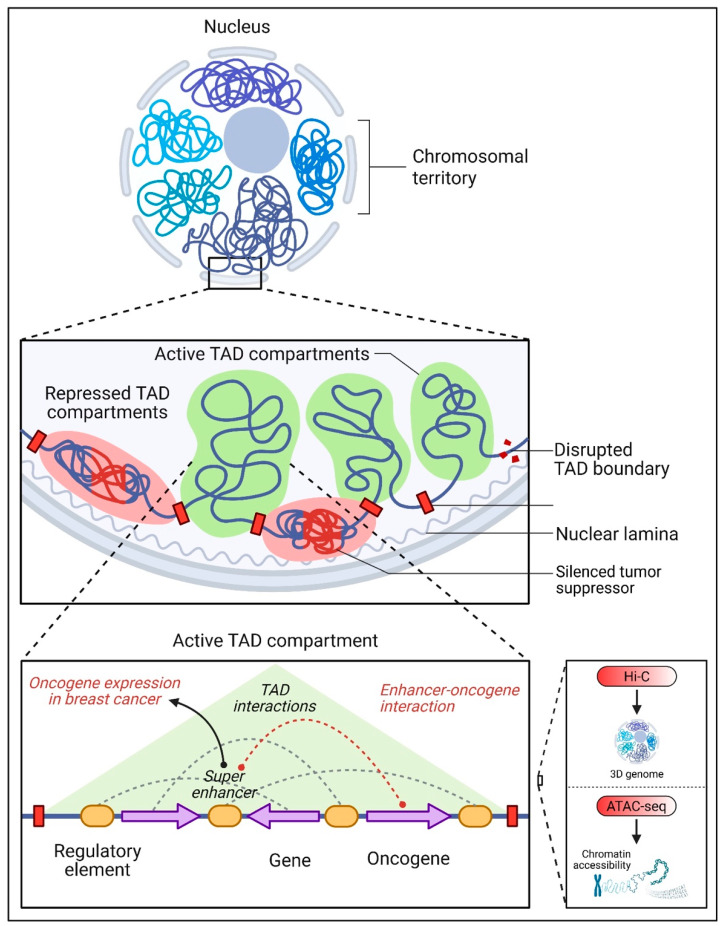
Chromatin architecture and its role in gene regulation and BC. The spatial organization of the genome within the nucleus is structured into chromosomal territories and subdivided into topologically associating domains (TADs). TADs are genomic regions with high internal chromatin interactions and are classified as either active compartments (green) or repressed compartments (pink). This 3D organization ensures precise enhancer–promoter interactions within the same TAD. In cancer, disrupted TAD boundaries may lead to the mislocalization of enhancers, resulting in aberrant oncogene activation or tumor suppressor silencing, as shown in the middle panel. The zoomed-in view depicts an active TAD compartment, highlighting super-enhancers that drive oncogene expression in BC through abnormal chromatin looping. On the right, tools like Hi-C (3D genome mapping) and ATAC-seq (chromatin accessibility profiling) are shown, which help decode genome organization and enhancer landscapes in cancer research.

The organization of the genome is a fundamental aspect of gene regulation. Chromatin looping and topologically associating domains (TADs) are key mechanisms that bring non-coding regulatory elements, such as enhancers, into physical proximity with their target genes, even when these elements are located far apart in the linear genome [71,72] (Figure 3). TADs are large genomic regions interacting more frequently with themselves than neighboring regions, creating insulated domains that help organize the genome into functional units. This spatial organization ensures precise regulation of gene expression by facilitating interactions between enhancers and promoters within the same TAD [73].

In BC, disruptions in chromatin architecture, such as alterations in TAD boundaries or chromatin looping, can lead to the misregulation of gene expression. TAD boundary rearrangements can cause enhancers to interact with oncogenes that they would not normally regulate, leading to their aberrant activation [74,75,76]. Conversely, tumor suppressor genes may be silenced if their regulatory elements are disrupted or relocated due to changes in chromatin organization. Such structural variations in the genome have been implicated in the progression and aggressiveness of BC, particularly in subtypes like TNBC [77].

Recent studies have highlighted the importance of chromatin accessibility and 3D genome organization in BC biology. Research has shown that super-enhancers—clusters of enhancers with exceptionally high activity—are often located within accessible chromatin regions and play a critical role in driving the expression of oncogenes in BC [78,79,80]. Disruption of these super-enhancers or their associated TADs can lead to gene overexpression in cell proliferation, survival, and metastasis. Moreover, advances in technologies such as Hi-C (a method to study 3D genome organization) and ATAC-seq (assay for transposase-accessible chromatin using sequencing) have provided unprecedented insights into how chromatin architecture is altered in cancer. These tools have revealed that BC cells often exhibit widespread changes in chromatin accessibility and TA organization compared to normal cells, underscoring the importance of these processes in tumorigenesis [81].

Targeting chromatin accessibility and 3D genome organization represents a promising therapeutic strategy in BC. inhibitors of chromatin remodelers or histone modifiers, such as BET inhibitors, have shown potential in preclinical studies by disrupting the activity of oncogenic enhancers and restoring normal gene expression patterns [82]. Additionally, understanding the role of TADs and chromatin looping in BC could lead to the development of novel biomarkers for diagnosis and prognosis.

### 3.2. Transcription Factor Recruitment

Non-coding DNA is far from being a passive bystander in the genome; it serves as a dynamic platform for recruiting transcription factors (TFs) and co-regulators that orchestrate gene expression. This process is particularly critical in cancer, where the dysregulation of transcription factor binding to non-coding regions can drive oncogenic pathways. In hormone receptor-positive BCs, estrogen receptor alpha (ERα) and androgen receptor (AR) bind to specific enhancer regions, activating transcriptional programs that promote tumor growth and survival [83]. These interactions highlight non-coding DNA’s pivotal role in shaping cancer cells’ transcriptional landscape.

The binding of transcription factors to non-coding regions is not random but is tightly regulated by the underlying DNA sequence and chromatin state. Enhancers and promoters, often marked by histone modifications such as H3K27ac and H3K4me3, provide accessible platforms for TF binding. However, mutations or epigenetic alterations in these regions can disrupt this finely tuned process. Single nucleotide polymorphisms (SNPs) or structural variations in enhancers can alter the binding affinity of TFs, leading to the aberrant activation or repression of target genes [84]. In BC, such disruptions have been linked to the overexpression of oncogenes like MYC or the silencing of tumor suppressors like BRCA1, underscoring the functional consequences of non-coding DNA alterations [85].

One of the most compelling examples of transcription factor recruitment in cancer is the role of ERα in luminal BC. ERα binds to thousands of enhancer regions across the genome, many in non-coding regions. These enhancers, often called estrogen response elements (EREs), drive gene expression in cell proliferation and survival. Recent studies have shown that the activity of these enhancers is not static but can be dynamically regulated by hormonal signals and epigenetic modifications. The depletion of ERα-bound enhancers has been shown to suppress tumor growth in preclinical models, highlighting their potential as therapeutic targets [86].

Similarly, the androgen receptor (AR) plays a critical role in BC, particularly in TNBC, where AR signaling can drive tumor progression. AR binds to enhancers and promoters in a ligand-dependent manner, activating transcriptional programs that promote cell survival and resistance to therapy. Recent research has revealed that AR can interact with non-coding RNAs, such as lncRNAs, to modulate its transcriptional activity. This crosstalk between TFs and non-coding elements adds another complexity to regulating gene expression in cancer [87].

The dysregulation of transcription factor recruitment is not limited to hormone receptors. Other TFs, such as NF-κB, STAT3, and AP-1, also rely on non-coding DNA for their function. In BC, the aberrant activation of these TFs has been linked to inflammation, immune evasion, and metastasis. NF-κB binds to enhancers that drive the expression of pro-inflammatory cytokines and chemokines, creating a tumor-promoting microenvironment. Targeting these interactions through small molecule inhibitors or epigenetic modulators represents a promising therapeutic strategy [82].

Despite these advances, several challenges remain. The non-coding genome’s complexity and regulatory networks make it difficult to predict the functional consequences of specific mutations or alterations. Moreover, the context-dependent nature of TF binding, influenced by cell type, developmental stage, and environmental cues, adds another layer of complexity. However, recent technological advancements, such as ChIP-seq, ATAC-seq, and CRISPR-based screens, provide unprecedented insights into non-coding DNA’s role in transcription factor recruitment and gene regulation [81].

## 4. Epigenetic Regulation by Non-Coding DNA

### 4.1. DNA Methylation

One of the well-characterized epigenetic modifications is DNA methylation, typically occurring at CpG islands within promoter regions. Hypermethylation of tumor suppressor gene promoters is a common event in BC, leading to their silencing and subsequent loss of tumor-suppressive functions. For instance, the hypermethylation of the *BRCA1* promoter has been linked to sporadic BCs, mimicking the loss-of-function mutations seen in hereditary cases [88]. Conversely, hypomethylation of oncogene promoters or enhancers can lead to their activation, driving tumor growth. Recent studies have shown that lncRNAs can guide DNA methyltransferases (DNMTs) to specific genomic regions, thereby modulating methylation patterns in a locus-specific manner [89]. This highlights the intricate interplay between ncRNAs and epigenetic machinery in cancer.

### 4.2. Histone Modifications

Histone modifications, such as acetylation and methylation, are another layer of epigenetic regulation influenced by non-coding DNA. lncRNAs like *HOTAIR* and *MALAT1* have been shown to recruit polycomb repressive complex 2 (PRC2) to specific genomic loci, leading to the trimethylation of histone H3 at lysine 27 (H3K27me3), a mark associated with gene silencing [90]. Similarly, miRNAs can indirectly influence histone modifications by targeting the mRNAs of chromatin-modifying enzymes. miR-101, which is frequently downregulated in BC, targets EZH2, a core component of PRC2. The loss of miR-101 leads to increased EZH2 activity, enhancing H3K27me3 levels and silencing tumor suppressor genes [91].

### 4.3. Non-Coding RNAs and Chromatin Remodeling

The role of ncRNAs in chromatin remodeling extends beyond histone modifications. They can also influence chromatin compaction and accessibility, thereby regulating the transcriptional activity of genes. The lncRNA *XIST*, known for its role in X-chromosome inactivation, has been implicated in BC progression by modulating chromatin structure and gene expression [51]. Additionally, miRNAs like miR-29b can target DNMTs, leading to global DNA hypomethylation and activation of oncogenic pathways [92]. These findings underscore ncRNAs’ multifaceted role in shaping cancer cells’ epigenetic landscape.

In addition to enhancers, lncRNAs play a critical role in regulating ER signaling and chromatin remodeling in luminal BCs. lncRNAs are transcripts longer than 200 nucleotides that do not encode proteins but function as regulators of gene expression. They can act as molecular scaffolds, decoys, or guides, influencing chromatin structure, transcription, and post-transcriptional regulation [93,94].

One of the well-characterized lncRNAs in luminal BC is HOTAIR (HOX transcript antisense RNA), which is frequently overexpressed in ER-positive tumors. HOTAIR interacts with the polycomb repressive complex 2 (PRC2) to silence tumor suppressor genes and promote tumor growth and metastasis [90]. In luminal BCs, HOTAIR has been shown to facilitate chromatin remodeling and transcriptional reprogramming by recruiting PRC2 to specific genomic loci, leading to the repression of genes involved in differentiation and apoptosis [43]. This reprogramming contributes to the aggressive phenotype of luminal tumors and their resistance to endocrine therapies.

Another lncRNA implicated in luminal BC is MALAT1 (metastasis-associated lung adenocarcinoma transcript 1), which regulates alternative splicing and gene expression. MALAT1 is overexpressed in luminal BCs and has been shown to enhance ERα-mediated transcription by acting as a scaffold for chromatin-modifying enzymes [48]. This interaction promotes the activation of cell proliferation and survival genes, making MALAT1 a potential therapeutic target for luminal BCs.

Epigenetic changes in non-coding regions are not only drivers of tumorigenesis but also contribute to therapeutic resistance. For example, in hormone receptor-positive BCs, epigenetic silencing of estrogen receptor alpha (ERα) target genes can lead to resistance to endocrine therapies like tamoxifen [95]. Similarly, in TNBC, epigenetic alterations in enhancer regions have been linked to resistance to chemotherapy and targeted therapies [96]. These observations highlight the clinical relevance of epigenetic regulation in BC and underscore the need for treatments that target these mechanisms.

### 4.4. Therapeutic Targeting of Epigenetic Regulators

Targeting epigenetic regulators represents a promising therapeutic strategy in BC. Drugs such as histone deacetylase inhibitors (HDAC inhibitors) and DNA methyltransferase inhibitors (DNMT inhibitors) have shown potential in preclinical and clinical studies by reversing aberrant epigenetic modifications and restoring normal gene expression patterns [97]. However, the complexity of epigenetic regulation and the interplay between different ncRNAs and chromatin-modifying enzymes pose significant challenges. Future research should focus on elucidating these interactions and developing combination therapies targeting multiple epigenetic regulation layers.

### 4.5. The Dynamic Interplay of m6A Epitranscriptomics and Cancer Progression

In the rapidly evolving field of oncology, a new paradigm is emerging that positions the epitranscriptome, and specifically N6-methyladenosine (m6A) RNA modifications, as a central regulatory force in carcinogenesis and therapeutic resistance. No longer considered a static post-transcriptional event, the m6A modification landscape is a dynamic and intricate system whose dysregulation can fundamentally drive oncogenesis. A particularly compelling mechanistic revelation is how seemingly benign genomic mutations—the synonymous mutations that do not alter the amino acid sequence of a protein—can profoundly impact tumor biology by disrupting m6A modification sites. Multiple systematic studies have now established a clear prevalence of these “silent” mutations within the m6A regulatory motifs across a wide spectrum of human cancers [98,99]. This discovery is not merely a scientific curiosity; it represents a tangible functional mechanism. For instance, the loss of an m6A site can directly compromise the stability of crucial tumor suppressor transcripts like CDKN2A and BRCA2, thereby fueling oncogenic processes without changing the protein’s coding sequence [100]. This is a powerful demonstration of a previously underappreciated layer of genetic regulation, where the inherited or somatic code itself remains intact, but the post-transcriptional destiny of its mRNA is irrevocably altered.

Moreover, the clinical ramifications of a dysregulated m6A epitranscriptome extend beyond tumor initiation, significantly impacting a tumor’s response to therapy. A striking example is seen in bladder cancer, where a reduction in m6A levels on transcripts such as SLC7A11 leads to enhanced mRNA stability and protein expression, ultimately conferring resistance to standard chemotherapy [101]. This provides a clear illustration of epitranscriptomic plasticity, a sophisticated survival mechanism through which cancer cells rapidly adapt to the selective pressures of therapeutic intervention. The intricate machinery governing m6A—comprising “writers” (methyltransferases that add the mark), “erasers” (demethylases that remove it), and “readers” (proteins that interpret the mark)—is also frequently imbalanced in cancer. Interestingly, the function of these regulatory proteins can be highly context-dependent, with the same player acting as an oncogene in one tumor type and a tumor suppressor in another [102]. This highlights the need for a nuanced, context-specific understanding of the m6A network. Beyond its genetic and therapeutic roles, the m6A epitranscriptome is also deeply intertwined with tumor metabolism, orchestrating the expression of genes involved in metabolic pathways to meet the high bioenergetic and anabolic demands of a rapidly proliferating cancer cell [103]. Collectively, these lines of inquiry converge to paint a compelling picture of m6A as a central organizing hub in cancer pathology. The paramount challenge now is to translate these detailed mechanistic insights into actionable therapeutic strategies that can specifically target and modulate the m6A machinery, thereby restoring cellular homeostasis and overcoming the formidable challenge of treatment resistance.

## 5. Relevance of Non-Coding RNA to BC

**Figure 4:**
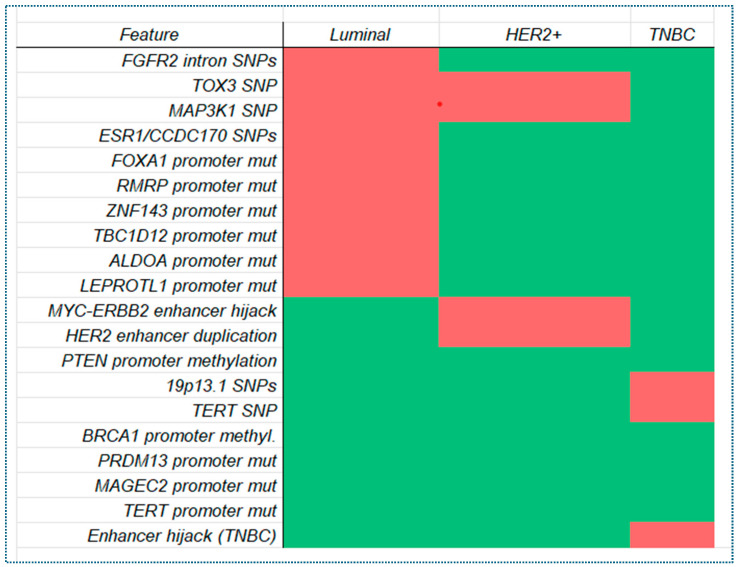
Heatmap of non-coding mutations across BC subtypes. The heatmap illustrates the presence of specific non-coding mutations across three major BC subtypes: Luminal, HER2+, and TNBC. Each row represents a unique regulatory mutation or region, while each column corresponds to a cancer subtype. Color intensity reflects the presence of mutations, with deeper red shades indicating confirmed involvement of that mutation in the corresponding subtype. Data were curated from peer-reviewed genomic studies, including germline variants, somatic promoter/enhancer mutations, and structural regulatory alterations linked to subtype-specific gene expression patterns and tumor progression.

BC is a highly heterogeneous disease, encompassing multiple molecular subtypes that exhibit distinct genetic, epigenetic, and transcriptomic profiles. These subtypes, including luminal A, luminal B, HER2-enriched, and TNBC, are driven by unique regulatory mechanisms that influence tumor behavior, treatment response, and patient outcomes (Figure 4). Among the key players in these regulatory networks are non-coding RNAs (ncRNAs), which include long non-coding RNAs (lncRNAs), microRNAs (miRNAs), and circular RNAs (circRNAs). These ncRNAs have emerged as critical regulators of gene expression, chromatin remodeling, and signaling pathways, making them central to the biology of BC subtypes. Understanding the role of ncRNAs in these subtypes is essential for unraveling BC’s molecular complexity and identifying subtype-specific biomarkers and therapeutic targets [104,105].

### 5.1. Non-Coding RNAs in Luminal Subtypes

Luminal A and luminal B BCs, which are hormone receptor-positive (HR+), account for the majority of BC cases [106,107]. These subtypes are characterized by the expression of estrogen receptor alpha (ERα) and/or progesterone receptor (PR), and hormone signaling pathways heavily influence their biology. ncRNAs play a pivotal role in modulating these pathways, often acting as fine-tuners of hormone receptor activity.

Similarly, MALAT1 has been shown to enhance ERα-mediated transcription by acting as a scaffold for chromatin-modifying enzymes, leading to the activation of genes involved in cell proliferation and survival [108]. These findings highlight the potential of targeting lncRNAs to disrupt hormone signaling in luminal BCs.

miRNAs also play a critical role in luminal subtypes by regulating key components of hormone signaling pathways. For example, miR-206 has been identified as a negative regulator of ERα expression, and its downregulation in luminal BCs contributes to the hyperactivation of ERα signaling [109]. Conversely, miR-375, which is upregulated in luminal B tumors, promotes cell proliferation by targeting the tumor suppressor gene RASD1 [110]. These miRNAs represent promising biomarkers for predicting treatment response and guiding therapeutic decisions in luminal BCs.

### 5.2. Non-Coding RNAs in HER2-Enriched Subtypes

HER2-enriched BCs are characterized by the amplification and overexpression of the HER2/ERBB2 gene, which drives aggressive tumor behavior [111,112]. ncRNAs have been shown to modulate HER2 signaling and contribute to the pathogenesis of this subtype. One notable example is the lncRNA BCAR4, which is upregulated in HER2-positive BCs and promotes tumor growth by activating the HER2 signaling pathway [113]. BCAR4 interacts with the transcription factors SMAD3 and FOXO3 to enhance gene expression in cell proliferation and survival, making it a potential therapeutic target for HER2-enriched BCs.

miRNAs also play a significant role in regulating HER2 signaling. miR-125a and miR-125b, which are frequently downregulated in HER2-positive BCs, act as tumor suppressors by targeting HER2 and other components of the HER2 signaling pathway [54]. Restoration of these miRNAs has inhibited tumor growth and sensitized HER2-positive BCs to targeted therapies such as trastuzumab [114]. These findings underscore the potential of miRNA-based therapies in HER2-enriched BCs.

### 5.3. Non-Coding RNAs in Triple-Negative BC (TNBC)

TNBC, which lacks expression of ERα, PR, and HER2, is the most aggressive and heterogeneous subtype of BC [115,116]. The absence of targeted therapies for TNBC underscores the need for a deeper understanding of its molecular drivers, many of which are regulated by ncRNAs [117].

lncRNAs such as HOTAIR and NEAT1 have been implicated in the pathogenesis of TNBC. HOTAIR, which is overexpressed in TNBC, promotes tumor growth and metastasis by silencing tumor suppressor genes and activating oncogenic pathways [118]. Similarly, NEAT1, which is upregulated in TNBC, enhances cell proliferation and survival by regulating the expression of genes involved in DNA repair and cell cycle progression [49]. These lncRNAs represent potential therapeutic targets for TNBC.

miRNAs also play a critical role in TNBC by regulating key signaling pathways such as the PI3K/AKT and NF-κB pathways. For example, miR-200c, which is frequently downregulated in TNBC, acts as a tumor suppressor by targeting ZEB1 and ZEB2, key regulators of epithelial-to-mesenchymal transition (EMT) [53]. Restoration of miR-200c has been shown to inhibit EMT and reduce tumor aggressiveness in TNBC [119]. Conversely, miR-221 and miR-222, which are upregulated in TNBC, promote tumor growth and therapy resistance by targeting the tumor suppressor gene PTEN [120]. These miRNAs represent promising biomarkers and therapeutic targets for TNBC.

### 5.4. Non-Coding RNAs as Biomarkers and Therapeutic Targets

The dysregulation of ncRNAs in BC subtypes has significant implications for the development of biomarkers and therapeutic strategies. For example, the expression levels of specific lncRNAs and miRNAs have been shown to correlate with tumor stage, grade, and patient outcomes, making them valuable prognostic and predictive biomarkers [121]. Additionally, the ability of ncRNAs to regulate key signaling pathways and modulate treatment response highlights their potential as therapeutic targets.

Several approaches are being explored to target ncRNAs in BC, including antisense oligonucleotides, small molecule inhibitors, and RNA-based therapies. Antisense oligonucleotides targeting HOTAIR have shown promise in preclinical studies by inhibiting tumor growth and metastasis in luminal and TNBC subtypes [122]. Similarly, small molecule inhibitors targeting the interaction between lncRNAs and chromatin-modifying enzymes are being developed as potential therapies for BC [123].

## 6. Non-Coding Elements in BC Subtypes

### 6.1. Luminal Subtypes (ER+/PR+)

Luminal BC, characterized by the expression of estrogen receptor (ER) and/or progesterone receptor (PR), represents the most common subtype of BC. These tumors are driven by estrogen signaling, which promotes cell proliferation and survival by activating ER target genes. However, the regulatory mechanisms underlying ER signaling are complex and involve coding genes and non-coding elements such as enhancers and lncRNAs. These elements play a critical role in shaping the transcriptional landscape of luminal BCs and contribute to the development of endocrine therapy resistance. Understanding the interplay between ER signaling, enhancers, and lncRNAs is essential for identifying novel therapeutic targets and improving outcomes for patients with luminal BC. Table 2 summarizes some key examples of non-coding mutations and their impacts on BC.

Luminal BC, characterized by the expression of estrogen receptor (ER) and/or progesterone receptor (PR), represents the most common subtype of BC. These tumors are driven by estrogen signaling, which promotes cell proliferation and survival by activating ER target genes. However, the regulatory mechanisms underlying ER signaling are complex and involve coding genes and non-coding elements such as enhancers and lncRNAs [157,158]. These elements are critical in shaping the transcriptional landscape of luminal BCs and contribute to developing endocrine therapy resistance [159]. Understanding the interplay between ER signaling, enhancers, and lncRNAs is essential for identifying novel therapeutic targets and improving outcomes for patients with luminal BC [160]. Table 3 represents some non-coding mutations, their location and pathogenicity.

In luminal BCs, ERα binds to specific enhancer regions, known as estrogen response elements (EREs), to regulate gene expression in cell proliferation, survival, and metabolism. The enhancer landscape of luminal BCs is highly dynamic and is shaped by the interplay between ERα, co-regulators, and chromatin-modifying enzymes [172]. Recent studies have shown that luminal BCs are associated with specific enhancer signatures that distinguish them from other BC subtypes. For example, super-enhancers—clusters of highly active enhancers—are frequently found near key ER target genes in luminal BCs [8]. These super-enhancers drive the high-level expression of genes involved in hormone signaling and cell cycle regulation, making them critical for the growth and survival of luminal tumors. However, mutations or epigenetic alterations in these enhancer regions can disrupt ER binding and lead to the dysregulation of ER target genes, contributing to endocrine therapy resistance [173]. One of the most well-studied examples of enhancer dysregulation in luminal BC is the mutation of the FOXA1 gene, which encodes a pioneer transcription factor that facilitates ERα binding to enhancers [174]. Similarly, mutations in the ESR1 gene, which encodes ERα, can create novel ER-binding sites that drive the expression of genes involved in therapy resistance [175]. These findings highlight the importance of enhancer integrity in maintaining ER signaling and response to therapy in luminal BCs.

### 6.2. HER2-Enriched Subtype

The HER2-enriched subtype of BC, defined by the amplification and overexpression of the HER2 (ERBB2) oncogene, represents approximately 15–20% of all BCs [176]. This subtype is characterized by aggressive tumor behavior, poor prognosis, and a reliance on HER2-driven signaling pathways for growth and survival [177]. While HER2-targeted therapies, such as trastuzumab and pertuzumab, have significantly improved patient outcomes, resistance to these therapies remains a major clinical challenge [178]. Emerging evidence suggests that non-coding elements, including enhancers, microRNAs (miRNAs), and mutations in regulatory regions, play a critical role in the overexpression of HER2 and the activation of downstream signaling pathways. Understanding the contribution of these non-coding elements to HER2 biology offers new insights into the mechanisms of HER2-driven tumourigenesis and resistance, as well as novel therapeutic opportunities [179].

#### 6.2.1. HER2 Amplification and Enhancer Activity

The amplification of the HER2 gene is a hallmark of the HER2-enriched subtype, but this amplification often extends to adjacent regulatory regions, including enhancers. Enhancers are distal regulatory elements that increase the transcription of target genes by interacting with promoters through chromatin looping. In HER2-amplified BCs, enhancers within the HER2 locus are co-amplified, leading to the overexpression of HER2 and its downstream signaling pathways. Recent studies have identified super-enhancers—clusters of highly active enhancers—near the HER2 gene in HER2-enriched BCs [8]. These super-enhancers drive the high-level expression of HER2 and other oncogenes, making them critical for the growth and survival of HER2-positive tumors. For example, the enhancer region upstream of HER2, known as the HER2-enhancer, has been shown to interact with the HER2 promoter through chromatin looping, facilitating its overexpression [180]. Disruption of this enhancer–promoter interaction through genetic or epigenetic means can reduce HER2 expression and inhibit tumor growth, highlighting the therapeutic potential of targeting enhancer activity in HER2-enriched BCs.

#### 6.2.2. miRNAs in HER2 Regulation and Signaling

miRNAs are small non-coding RNAs that regulate gene expression by binding target mRNAs’ 3′ untranslated regions (UTRs), leading to their degradation or translational repression. In HER2-enriched BCs, miRNAs play a dual role in regulating HER2 expression and modulating downstream signaling pathways. One of the most well-studied miRNAs in HER2-positive BC is miR-21, which is frequently overexpressed in this subtype. miR-21 promotes HER2 signaling by targeting tumor suppressor genes such as PTEN and PDCD4, which negatively regulate the PI3K/AKT and MAPK pathways [181]. By silencing these tumor suppressors, miR-21 enhances the activation of HER2-driven signaling pathways, contributing to tumor growth and therapy resistance. Inhibition of miR-21 has been shown to restore sensitivity to HER2-targeted therapies in preclinical models, suggesting that targeting miR-21 could be a promising therapeutic strategy [52]. In contrast, some miRNAs act as tumor suppressors by directly targeting HER2. For example, miR-125a and miR-125b, which are frequently downregulated in HER2-positive BCs, inhibit HER2 expression by binding its 3′ UTR [182]. Restoration of miR-125a and miR-125b has been shown to reduce HER2 levels and inhibit tumor growth, highlighting their potential as therapeutic agents. Similarly, miR-205, which is downregulated in HER2-positive BCs, targets HER2 and other components of the HER2 signaling pathway, making it another potential candidate for miRNA-based therapies [56].

#### 6.2.3. Non-Coding Mutations in HER2 Regulatory Regions

In addition to enhancers and miRNAs, non-coding mutations in regulatory regions of HER2 or its co-expressed genes can contribute to HER2 overexpression and therapy resistance. These mutations can alter the binding of transcription factors, disrupt enhancer–promoter interactions, or create novel regulatory elements that drive HER2 expression. For example, mutations in the HER2 promoter region have been identified in HER2-positive BCs and are associated with increased HER2 expression and poor prognosis [183]. These mutations can create new binding sites for transcription factors or disrupt the binding of repressors, leading to the dysregulation of HER2 expression. Similarly, mutations in enhancer regions near HER2 can alter their activity, resulting in the overexpression of HER2 and other oncogenes [184]. Non-coding mutations can also affect the expression of genes co-amplified with HER2, such as GRB7 and STARD3, which are located in the same genomic region. These genes are often co-expressed with HER2 and contribute to HER2-driven tumourigenesis. Mutations in the regulatory regions of these genes can enhance their expression and further promote tumor growth and therapy resistance [185]. Targeting these non-coding mutations through gene editing or epigenetic modulation represents a promising therapeutic strategy for HER2-enriched BCs.

### 6.3. TNBC and Basal-Like (BLBC) Subtype

#### 6.3.1. miRNAs in TNBC/BLBC: The miR-200 Family and EMT

miR-21 promotes tumor growth and chemoresistance [2,186]. miR-34a targets genes involved in cell cycle regulation and apoptosis [187]. The miR-200 family, which includes miR-200a, miR-200b, miR-200c, miR-141, and miR-429, is frequently downregulated in BLBCs. These miRNAs act as tumor suppressors by targeting ZEB1 and ZEB2, key regulators of epithelial-to-mesenchymal transition (EMT) and stemness [188]. The downregulation of the miR-200 family in BLBCs leads to the overexpression of ZEB1 and ZEB2, promoting EMT, stemness, and metastasis [189]. Restoration of miR-200 family members has been shown to inhibit EMT and reduce tumor aggressiveness in BLBCs, highlighting their potential as therapeutic agents [190].

#### 6.3.2. Immune Evasion and Therapeutic Opportunities

One of the hallmarks of BLBCs is their ability to evade immune surveillance, which contributes to their aggressive behavior and resistance to therapy. Non-coding elements play a critical role in regulating immune evasion in BLBCs by modulating the expression of immune checkpoint molecules and other factors involved in the tumor-immune interaction. The lncRNA PVT1, which is frequently overexpressed in BLBCs, promotes immune evasion by upregulating the expression of PD-L1, a key immune checkpoint molecule. PVT1 interacts with the transcription factor MYC to enhance PD-L1 expression, enabling BLBC cells to evade immune detection. Inhibition of PVT1 has been shown to reduce PD-L1 levels and enhance immune-mediated tumor killing, suggesting that targeting PVT1 could be a promising strategy for immunotherapy in BLBCs [50]. Similarly, miRNAs such as miR-200c and miR-34a have been shown to regulate immune evasion in BLBCs by targeting genes involved in the PD-1/PD-L1 pathway [190]. Restoration of these miRNAs has enhanced immune-mediated tumor killing and sensitized BLBC cells to immune checkpoint inhibitors. These findings highlight the potential of targeting ncRNAs to overcome immune evasion and improve the efficacy of immunotherapy in BLBCs.

The basal-like subtype of BC is characterized by distinct chromatin landscapes and non-coding RNA profiles that drive tumor aggressiveness, stemness, and immune evasion [191,192]. Enhancers and lncRNAs such as HOTAIR, MALAT1, and NEAT1 regulate genes involved in stemness and therapy resistance [193,194], while miRNAs such as the miR-200 family and miR-34a modulate EMT and immune evasion. Targeting these non-coding elements represents a promising therapeutic strategy for overcoming therapy resistance and improving outcomes for patients with BLBC [195,196]. By unraveling the complex interplay between non-coding elements and the biology of BLBCs, we can develop more effective therapies that address the root causes of this aggressive subtype.

## 7. Therapeutic Challenges and Future Directions in BC Research

### 7.1. General Challenges and Future Directions

The functional redundancy and context-dependent nature of non-coding RNAs (ncRNAs) complicate their therapeutic targeting. Additionally, the delivery of RNA-based therapies to tumor cells remains a significant hurdle [197]. Future research should focus on elucidating the complex regulatory networks involving ncRNAs and developing innovative delivery systems to overcome these challenges [198]. In conclusion, ncRNAs are central to the biology of BC subtypes, regulating key signaling pathways and modulating treatment response. Their dysregulation in luminal, HER2-enriched, and TNBC subtypes highlights their potential as biomarkers and therapeutic targets [199]. By unraveling the complex interplay between ncRNAs and molecular pathways, we can develop more effective therapies that improve outcomes for BC patients.

### 7.2. Endocrine Therapy Resistance: Mechanisms and Implications in Luminal BC

Endocrine therapies, such as tamoxifen and aromatase inhibitors, are the cornerstone of treatment for luminal BCs [200,201]. These therapies target estrogen receptor (ER) signaling by blocking ERα activity or reducing estrogen levels. However, a significant proportion of patients develop resistance to these therapies, leading to disease progression and poor outcomes. The mechanisms underlying endocrine therapy resistance are complex and involve genetic and epigenetic alterations. Mutations in the ESR1 gene, which encodes ERα, have been identified as a key mechanism of resistance, occurring in approximately 30% of hormone receptor-positive metastatic BC cases [202].

Upregulation of growth factor receptor pathways, such as the PI3K/AKT/mTOR signaling cascade, has been implicated in diminishing the efficacy of endocrine treatments [203]. Epigenetic modifications, including DNA methylation and histone acetylation, also contribute to therapy resistance by altering gene expression patterns critical for ER signaling [204]. Furthermore, the involvement of BC stem cells has been recognized as a factor in mediating recurrence and resistance to anti-estrogen therapies [205].

One of the key mechanisms of resistance is the dysregulation of enhancers and ER-binding sites. Mutations in the ESR1 gene, common in endocrine-resistant luminal BCs, can create novel ER-binding sites that drive the expression of genes involved in therapy resistance [159]. Similarly, alterations in the enhancer landscape, such as the loss of FOXA1 binding or the gain of super-enhancers near oncogenes, can lead to the activation of alternative signaling pathways that bypass ER dependence [206].

lncRNAs also play a critical role in endocrine therapy resistance. For example, HOTAIR has been shown to promote resistance to tamoxifen by silencing tumor suppressor genes and activating oncogenic pathways [207]. Similarly, MALAT1 enhances gene expression in cell survival and proliferation, contributing to therapy resistance [208]. These findings underscore the importance of targeting lncRNAs to overcome endocrine therapy resistance in luminal BCs.

### 7.3. Therapeutic Opportunities and Future Directions in Luminal BC

The dysregulation of enhancers and lncRNAs in luminal BCs presents unique therapeutic opportunities. Targeting enhancer activity could restore normal ER signaling and sensitize tumors to endocrine therapies. Small molecule inhibitors of enhancer-associated proteins, such as BET inhibitors, have shown promise in preclinical studies by disrupting super-enhancers’ activity and reducing oncogenes’ expression [209]. Similarly, targeting lncRNAs such as HOTAIR and MALAT1 could reverse chromatin remodeling and transcriptional reprogramming, restoring sensitivity to endocrine therapies.

Several approaches are being explored to target lncRNAs in luminal BCs, including antisense oligonucleotides, small molecule inhibitors, and RNA-based therapies. For instance, antisense oligonucleotides targeting HOTAIR have shown efficacy in preclinical models by inhibiting tumor growth and metastasis [122]. Similarly, small molecule inhibitors targeting the interaction between lncRNAs and chromatin-modifying enzymes are being developed as potential therapies for luminal BCs [123].

Luminal BCs are driven by estrogen receptor signaling, which is tightly regulated by enhancers and lncRNAs [210,211,212]. The enhancer landscape of luminal tumors is highly dynamic and is shaped by the interplay between ERα, co-regulators, and chromatin-modifying enzymes. Mutations or epigenetic alterations in enhancers and ER-binding sites can lead to the dysregulation of ER target genes and contribute to endocrine therapy resistance [121,213,214]. Similarly, lncRNAs such as HOTAIR and MALAT1 are critical in chromatin remodeling and transcriptional reprogramming, promoting tumor growth and therapy resistance [122,215,216]. Targeting these non-coding elements represents a promising therapeutic strategy for overcoming endocrine therapy resistance and improving outcomes for patients with luminal BC.

## 8. Conclusions

The dysregulation of ncRNAs in BC underscores their critical role in tumorigenesis, metastasis, and therapy resistance. lncRNAs such as HOTAIR and MALAT1 drive chromatin remodeling and transcriptional reprogramming, contributing to the aggressive behavior of luminal and TNBC subtypes. miRNAs, including the miR-200 family and miR-21, regulate key signaling pathways such as EMT, HER2 signaling, and immune evasion, making them promising therapeutic targets. circRNAs and snoRNAs, though less studied, are emerging as important regulators of gene expression and RNA processing, with potential implications for biomarker development and targeted therapies. piRNAs, a relatively new class of ncRNAs, are also gaining attention for their role in epigenetic regulation and tumor progression. The integration of ncRNA profiling into clinical practice could revolutionize the diagnosis, prognosis, and treatment of BC. For example, targeting HOTAIR or MALAT1 with antisense oligonucleotides could modulate chemoresistance in TNBC, while restoring miR-200 family expression could inhibit metastasis in basal-like subtypes. Furthermore, developing circRNA-based biomarkers could improve the early detection of HER2-positive BCs and predict responses to therapy. By unraveling the complex regulatory networks involving ncRNAs, we can develop more effective treatments that address the root causes of BC heterogeneity and resistance.

## Figures and Tables

**Table 2:** Key examples of non-coding mutations and their impacts in BC.

Non-Coding Region	Mutation Type	Associated Gene/Pathway	Functional Impact	BC Subtype	Clinical Relevance	References
**Enhancer**	SNP	MYC	Increased MYC expression	Triple-negative	Potential drug target	[124,125]
**Promoter**	Deletion	TP53	Reduced gene expression	Luminal	Diagnostic biomarker	[23,126,127]
**lncRNA**	Overexpression	MALAT1	Promotes metastasis	HER2+	Prognostic indicator	[23,128,129]
**miRNA**	Mutation	miR-21	Disrupted mRNA regulation	All subtypes	Therapeutic target (anti-miR)	[130,131,132]
**Intronic Region**	Insertion	BRCA1	Aberrant splicing	Hereditary cases	Risk prediction	[133,134]
**3′ UTR**	SNP	CDKN1A	Alters miRNA binding	Triple-negative	Regulatory variant affecting cell cycle	[135,136]
**5′ UTR**	Methylation	FOXA1	Transcriptional repression	Luminal A	Epigenetic marker of prognosis	[137]
**Circular RNA**	Overexpression	circHIPK3	Sponges tumor suppressor miRNAs	TNBC	Modulates chemoresistance	[138]
**Pseudogene**	Amplification	PTENP1	Competes for miRNA binding	HER2-	Regulates tumor suppressor PTEN	[139,140,141]
**Intergenic**	SNP	8q24 locus	Modulates enhancer–promoter looping	ER+	Associated with familial risk	[142,143]
**Ultraconserved Region**	SNP	TUC338	Enhances oncogenic signaling	TNBC	Putative diagnostic biomarker	[144,145]
**Antisense RNA**	Overexpression	ANRIL	Silences CDKN2A/B via PRC2 recruitment	Luminal B	Epigenetic driver of proliferation	[146,147,148]
**Transcribed Spacer**	Insertion	DLEU2	Affects miRNA cluster stability	HER2+	miRNA network regulator	[149,150]
**Repeat Element**	Expansion	LINE-1	Induces genomic instability	Basal-like	Potential target for genome stabilization	[151,152,153]
**Bidirectional Promoter**	Deletion	ESR1/GREB1	Alters co-regulated gene expression	ER+	Estrogen response marker	[154,155,156]

**Table 3:** Key examples of non-coding mutations, their location, and pathogenicity.

Non-Coding Mutation	Location	Nucleotide Change	Pathogenicity/Significance	Reference
*TERT* promoter	Chromosome 5p15.33	C228T or C250T	The mutations create a new binding site for ETS/TCF transcription factors, leading to the overexpression of telomerase, a key hallmark of cancer, which promotes unlimited cell proliferation. While rare in breast cancer, these mutations are considered pathogenic.	[161]
*MYC* distal enhancer	Chromosome 8q24	Point mutations and SNPs (e.g., rs13281615)	Mutations in this region, which is ~2 Mb from the *MYC* gene, can increase the risk of breast cancer. They are believed to alter the binding of transcription factors, leading to the deregulation and overexpression of the MYC oncogene.	[162,163,164]
*PIM1* super-enhancer	Chromosome 6p21.2	Various mutations	Mutations within the super-enhancer of the *PIM1* oncogene, particularly in luminal breast cancer, are linked to increased *PIM1* expression. This is often associated with a more aggressive phenotype and poorer prognosis.	[165,166,167,168]
miR-126 promoter	Chromosome 3p21	DNA methylation	While not a direct mutation, hypermethylation of the miR-126 promoter silences its expression. Since miR-126 acts as a tumor suppressor, its loss promotes metastasis and is associated with poor prognosis in breast cancer patients.	[169,170,171]

## Data Availability

All data generated are presented in the current MS.

## Abbreviations

**AR**; androgen receptor; **ATAC-seq**; assay for transposase-accessible chromatin using sequencing; **BC**; Breast cancer; **BCAR4**; BCAR4; **BET**; bromodomain and extraterminal; **BRCA1**; BC type 1 susceptibility protein; **circRNAs**; circular RNAs; **ChIP-seq**; chromatin immunoprecipitation sequencing; **DNMT**; DNA methyltransferase; **EMT**; epithelial-to-mesenchymal transition; **ER**; estrogen receptor; **ERα**; estrogen receptor alpha; **ERBB2**; erb-b2 receptor tyrosine kinase^1^ 2; **ERE**; estrogen response element; **EZH2**; enhancer of zeste homolog 2; **FOXA1**; forkhead box A1; **FOXO3**; forkhead box O3; **H3K27ac**; histone H3 lysine 27 acetylation; **H3K27me3**; histone H3 lysine 27 trimethylation; **H3K4me3**; histone H3 lysine 4 trimethylation; **HDAC**; histone deacetylase; **HER2**; human epidermal growth factor receptor 2; **Hi-C**; high-throughput chromosome conformation capture; **HOTAIR**;^2^ HOX transcript antisense RNA; **HR+**; hormone receptor-positive; **lncRNAs**; long non-coding RNAs; **MALAT1**; metastasis-associated lung adenocarcinoma transcript 1; **miRNAs**; microRNAs; **MYC**; myelocytomatosis oncogene; **NEAT1**; nuclear paraspeckle assembly transcript 1; **NF-κB**; nuclear factor kappa B; **ncRNAs**; non-coding RNAs; **PDCD4**; programmed cell death 4; **PD-L1**; programmed death-ligand 1; **piRNAs**; Piwi-interacting RNAs; **PR**; progesterone receptor; **PRC2**; polycomb repressive complex 2; **PTEN**; phosphatase and tensin homolog; **PVT1**; plasmacytoma variant translocation 1; **RNA**; ribonucleic acid; **RASD1**; RASD family member 1; **SMAD3**; SMAD family member 3; **snoRNAs**; small nucleolar RNAs; **SNHG1**; small nucleolar RNA host gene 1; **SNPs**; single nucleotide polymorphisms; **STAT3**; signal transducer and activator of transcription 3; **TADs**; topologically associating domains; **TNBC**; triple-negative breast cancer; **TP53**; tumor protein p53; **UTR**; untranslated region; **XIST**; X inactive specific transcript; **ZEB1**; zinc finger E-box binding homeobox 1; **ZEB2**; zinc finger E-box binding homeobox 2.
